# An improved method with a wider applicability to isolate plant mitochondria for mtDNA extraction

**DOI:** 10.1186/s13007-015-0099-x

**Published:** 2015-12-21

**Authors:** Zaheer Ahmed, Yong-Bi Fu

**Affiliations:** Plant Gene Resources of Canada, Saskatoon Research and Development Centre, Agriculture and Agri-Food Canada, 107 Science Place, Saskatoon, SK S7N 0X2 Canada

**Keywords:** Mitochondria, mtDNA, Isolation method, Wheat tissues, Crop species

## Abstract

**Background:**

Mitochondria perform a principal role in eukaryotic cells. Mutations in mtDNA can cause mitochondrial dysfunction and are frequently associated with various abnormalities during plant development. Extraction of plant mitochondria and mtDNA is the basic requirement for the characterization of mtDNA mutations and other molecular studies. However, currently available methods for mitochondria isolation are either tissue specific or species specific. Extracted mtDNA may contain substantial chloroplast DNA (cpDNA) and nuclear DNA (nDNA) and its end use efficiency can be reduced. Clearly, an effective mitochondria isolation method is warranted with wider applicability and with minimum contamination from cpDNA and nDNA.

**Results:**

Here we reported an improved method for isolating mitochondria from dry wheat seeds and its extension to dead seeds, viable seeds, etiolated leaf tissue and several other plant species: oat, Arabidopsis, flax, and yellow mustard. The isolated mitochondria were successfully used to extract mtDNA with QIAamp DNA mini kit (Qiagen). The extracted mtDNA from the assayed samples of these species was intact in large quantity and showed little contamination from nDNA, cpDNA, RNA, and proteins. The mtDNA extracted from dead wheat seeds was also substantial, but more degraded and less intact when compared to those from viable seeds and other tissues.

**Conclusion:**

The improved method was successfully applied to isolate mitochondria and extract mtDNA from several different tissues and plant species. The major advance in the improvement lies in its wider application with the same mitochondria extraction medium to different tissues and species. The improvement is significant, as it helps to widen the scope of future plant mitochondria research.

**Electronic supplementary material:**

The online version of this article (doi:10.1186/s13007-015-0099-x) contains supplementary material, which is available to authorized users.

## Background

Mitochondria perform an important role in eukaryotic cells, including the generation of chemical energy in the form of ATP, compartmentalization of cellular metabolism, and regulation of various physiological processes [1, 2]. Mitochondria possess their own genetic system, including multiple copies of the mitochondrial DNA (mtDNA) and the machinery required for mtDNA replication and translation [2]. mtDNA encodes necessary proteins for ATP production, plays a significant role in plant development and reproduction, and exhibits a different parental inheritance from other organelles [3]. Mutations in mtDNA can cause mitochondrial dysfunction and are frequently found to be associated with various abnormalities during plant development [4, 5].

The ability to isolate intact and pure mitochondria from different plant species and tissues is a key for a functional analysis of plant mitochondria and non-functional assay of mtDNA mutations. Intact mitochondria are required for isolating mitochondria proteins, measuring mitochondria function, and extracting mtDNA. Different protocols for isolating functional mitochondria have been reported in different crop species and plant cell culture: Arabidopsis [6–10], pea [11, 12], wheat [13] or plant cell culture [14]. Protocols for extracting proteins [15–17] and mtDNAs [18–21] form different crop species also exist for non-functional analysis of mitochondria. However, issues associated with these protocols are not lacking and the impurity of isolated mitochondria is the major concern, particularly for a non-functional analysis [22–24]. Extracting proteins or mtDNA from an impure isolation of mitochondria is less effective for the downstream physiological and genomic analyses.

A unique feature of the reported isolation protocols is the specificity in their applications, as these methods were usually developed either for specific application to a single plant species or based on specific tissue of a plant species. This is understandable, as different plant species or tissues usually possess variable phenolic compounds or different metabolite profiles and these biochemical substances can easily damage the integrity of mitochondrial membranes. Such variations also help to explain why different mitochondria isolation medium compositions and centrifugation speed and time are required to make an mtDNA extraction more effective. This requirement in turn, however, poses limits for the application of the published methods to plant species different from those specified. Also, some earlier methods [e.g., see 8, 12, 14] require a preparation of continuous or discontinuous density gradient, from which intact mitochondria are collected. Such mitochondria collection carries a large risk of contamination from other organelles such as chloroplasts, nuclei, and peroxisomes. Consequently, mtDNA extractions are not always effective and the extracted DNAs are often contaminated with chloroplast DNA (cpDNA) and nuclear DNA (nDNA). The variable purity of mtDNA extraction makes various downstream applications, particularly high-throughput sequencing and molecular genetic analysis, less effective.

For more effective non-functional application of isolated mitochondria, we made an attempt to improve existing methods with the goal to isolate pure mitochondria from different tissues of the same plant and also from different plant species to enhance mtDNA extraction with minimum contamination from cpDNA and nDNA. We focused on two previously published protocols [16, 25], and came up with a single mitochondria extraction medium that can be used for mitochondria isolation from various wheat (*Triticum aestivum*) tissues and seeds of different plant species (cultivated oat, *Avena sativa*; Arabidopsis, *Arabidopsis thaliana*; cultivated flax, *Linum usitatissimum*; and yellow mustard, *Sinapis alba*), although with minor modifications. Briefly, the improvement used a single Percoll density gradient to remove chloroplasts and nuclei as opposed to previous methods with continuous or discontinuous density gradients risking the contamination with nuclei and chloroplasts. We also successfully extended our improved method to isolate mitochondria and extract mtDNA from physiologically dead seeds generated from an accelerated ageing test (AAT). Our efforts have generated an improved method with a wider application to isolate mitochondria from different species and tissues and opened new opportunities to study mtDNA mutations of viable and dead seeds.

## Results

### The improved method for mitochondria isolation and its extensions

**Fig. 1:**
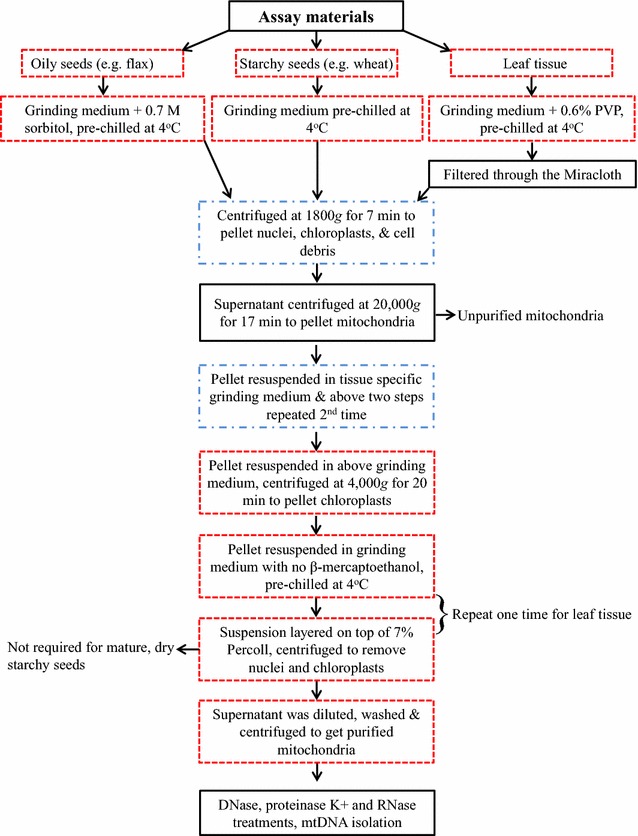
Flow chart of the improved method for isolating mitochondria in plant species. The improved method was based on two previously published protocols [16, 25]. The original (*black boxes*), new (*red dashed boxes*), and modified (*blue dash-dot boxes*) steps are shown

Our improved method was made based on two published protocols [16, 25] to isolate mitochondria from dry wheat seeds. The improvement involved various steps in the original methods typically associated with ultra and gradient centrifugations to generate pure mtDNA. The major advance was the use of a single Percoll density gradient, rather than the continuous or discontinuous density gradients, to remove chloroplast and nuclei. With the successful improvement, the method was further extended to the application of different seed and leaf tissues such as dead seed, viable seeds, etiolated leaf tissue, and other oily seed species. For other assayed tissues, some additional improvements were also made in the grinding medium. For example, polyvinylpyrrolidone (PVP) was added to adsorb phenolic compounds from leaf tissue. Sorbitol was added to separate mitochondria from spherosomes in oily seeds. These improvements made the mitochondria isolation more effective from a range of tissue types and species with minimal contamination from other organelles. Figure 1 shows the major procedures of the improved method with an illustration of the original, new, and modified steps for isolating plant mitochondria.

### Evaluation with dry wheat seeds

**Fig. 2:**
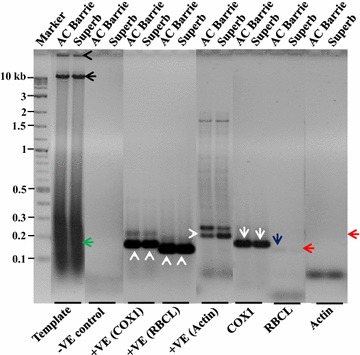
Integrity and purity tests of mtDNA extracted from dry starchy seeds of two wheat genotypes. For integrity test ~28 µg of purified mtDNA was loaded on to the gel, showing in Template. For purity test 120 ng of mtDNA was used for each PCR reaction with specific mitochondria, chloroplast and nuclear primers (COX1, RBCL and actin, respectively). −VE indicates PCR with no template with the actin primers. +VE indicates unpurified mtDNA used as template with three different sets of primers. The purity based on three specific primer sets is also shown. To illustrate, *black arrow head* is also used to show mtDNA stacked in the wells; *black arrow*, intact mtDNA; a *green arrow*, degraded mtDNA; *white arrow* heads, target products with +VE controls; *white arrows*, target product with COX1; *red arrows*, target product with RBCL and actin; *a blue arrow*, target amplification from residual template with RBCL primers. Names of the genotypes corresponding to each reaction are represented at the top, and DNA ladder in kb is represented on the left side of the gel

The improved method was first tested with two wheat genotypes, AC Barrie and Superb. The isolated mitochondria were successfully applied to extract mtDNA, and the extracted mtDNA displayed better quality and large quantity (Fig. 2; Table 1). The high purity was observed with no or minimal contamination from nDNA, cpDNA, RNA, proteins and other impurities. Also, the mtDNA extractions yielded 400 ng/µl or more mtDNA from a single preparation with a final volume of 40–50 µl. To assess the integrity, the purified mtDNA (~28 µg) was loaded onto the agarose gels. The gels showed that intact mtDNAs were obtained (Fig. 2, black arrow), but some degraded mtDNA were also revealed (Fig. 2, green arrow). Such degradation may have occurred during handling and storage.

**Table 1:** Results of the quantification and quality analyses of mtDNA extracted from different seed and leaf tissues of five different plant species

Sample	PicoGreen ng/µl	NanoDrop ng/µl	A_260_/A_280_	A_260_/A_230_
AC Barrie	34.33	625	1.83	2.08
Superb	33.88	606	1.88	2.09
Dead seed B	27.02	410	1.94	1.93
Dead seed S	27.43	413	2.01	2.00
AS B	29.69	530	1.91	2.00
AS S	30.93	513	1.97	2.08
AL B	28.83	493	1.90	2.02
AL S	28.33	491	1.95	2.09
NS B	29.57	634	1.83	2.03
NS S	27.88	620	1.85	2.06
NL B	27.22	471	1.92	2.05
NL S	26.16	485	1.84	2.03
Oat	34.38	617	1.94	2.04
Arabidopsis	30.62	544	1.89	2.11
Mustard	30.19	484	1.85	2.00
Flax	31.28	494	1.93	1.98

A mtDNA sample was measured for both quantity and quality by NanoDrop, diluted to 40 ng/µl and confirmed for quantity by PicoGreen

*AS* abnormally germinated seed tissue, *AL* abnormally germinated leaf tissue, *NS* normally germinated seed tissue, *NL* normally germinated leaf tissue

Sample names with B or S represent wheat genotypes AC Barrie or Superb, respectively

The purity of extracted mtDNA was confirmed from several PCR assessments using specific primers of mtDNA gene cytochrome C oxidase I (COX1), cpDNA gene rubisco-large subunit (RBCL), and nDNA gene actin. We also considered a positive control (unpurified mtDNA) with these primers and a negative control (no mtDNA) with the actin primer only. With the negative control, we did not detect any amplification (Fig. 2), which confirmed that there was no exogenous genomic DNA contamination that could interfere with the PCR analyses. The assessments with positive controls (Fig. 2, white arrow heads) showed that all the primers (COX1, RBCL, and actin) successfully amplified the target products (178, 158, and 206 bp), respectively. However, multiple bands were also observed in the positive control with the actin primers; some of which may be PCR artefacts. After the confirmation of the gene specificity with the assayed primers, cpDNA and nDNA contaminations were determined using the purified mtDNA as template. The COX1 primers successfully amplified the target product in both wheat genotypes (Fig. 2, white arrow). The RBCL and actin primers showed a very faint band of the target products (Fig. 2, red and blue arrows), confirming the purified mtDNA had little cpDNA and nDNA contamination.

### Evaluation with different tissues and species

**Fig. 3:**
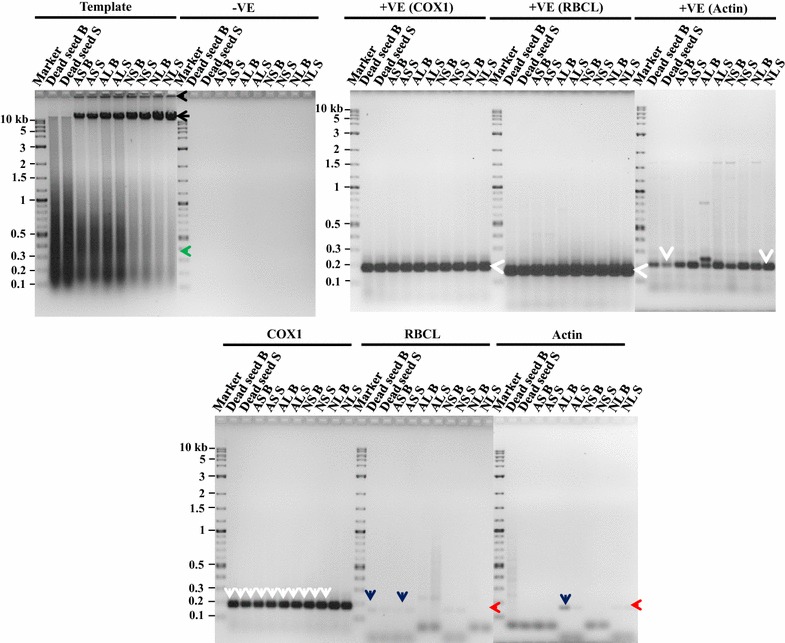
Integrity and purity tests of mtDNA extracted from five seed and leaf tissues of two wheat genotypes. For integrity test ~28 µg of purified mtDNA was loaded on to the gel, showing in Template. For purity test 120 ng of mtDNA was used for each PCR reaction with specific mitochondria, chloroplast and nuclear primers (COX1, RBCL and actin, respectively). *−VE* indicates PCR with no template with the actin primers. *+VE* indicates unpurified mtDNA used as template with three different sets of primers. The purity based on three specific primer sets is also shown. To illustrate, *black arrow head* is also used to show mtDNA stacked in the wells; *black arrow*, intact mtDNA; a *green arrow*, degraded mtDNA; *white arrow heads*, target products with +VE controls; *white arrows*, target product with COX1; *red arrows*, target product with RBCL and actin; *blue arrows*, target amplification from residual template with RBCL or actin primers. Names of the seed and leaf tissues corresponding to each reaction are represented at the top, and DNA ladder in kb is represented on the left side of the gel. *AS* abnormally germinated seed tissue, *AL* abnormally germinated leaf tissue, *NS* normally germinated seed tissue, *NL* normally germinated leaf tissue. Sample names with *B* or *S* represent wheat genotypes AC Barrie or Superb, respectively

**Fig. 4:**
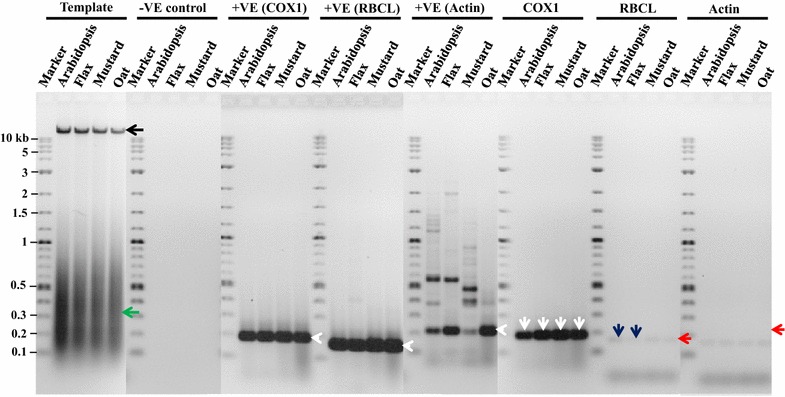
Integrity and purity tests of mtDNA extracted from dry seeds of four other plant species. For integrity test ~28 µg of purified mtDNA was loaded on to the gel, showing in Template. For purity test 120 ng of mtDNA was used for each PCR reaction with specific mitochondria, chloroplast and nuclear primers (COX1, RBCL and actin, respectively). −*VE* indicates PCR with no template with the actin primers. +*VE* indicates unpurified mtDNA used as template with three different sets of primers. The purity based on three specific primer sets is also shown. To illustrate, *black arrow* is also used to show intact mtDNA; *a green arrow*, degraded mtDNA; *white arrow heads*, target products with +VE controls; *white arrows*, target product with COX1; *red arrows*, target product with RBCL and actin; *blue arrows*, target amplification from residual template with RBCL. Names of the plant species corresponding to each reaction are represented at the top, and DNA ladder in kb is represented on the left side of the gel

The assessments on various extensions of the improved method to dead seeds, viable seeds, leaf tissue and seeds of different species were also fruitful. The isolated mitochondria from these tissues were also successfully applied to extract mtDNA. The integrity of the purified mtDNA from all these samples was observed by loading ~28 µg of the purified mtDNA onto the agarose gels. Specifically, the intact mtDNA from all samples were found (Figs. 3, 4, black arrows). However, the amount of the intact mtDNA as visible by band intensity was lower in dead seed and higher in normal seed and leaf tissues (Fig. 3, black arrow) and vice versa for mtDNA degradation (Fig. 3, green arrow). In some samples (AS, AL, NS, and NL), mtDNA stacking in the wells was also observed when compared to dead seeds in which mtDNA stacking was absent (Fig. 3, black arrow head).

The results of all the assayed samples of different tissues and species with negative (Figs. 3, 4) and positive (Figs. 3, 4, white arrow heads) controls were similar to the findings with dry wheat seeds (Fig. 2). For all these samples, mtDNA purity was also tested using the same gene-specific primers with the purified mtDNA as template. The COX1 primers successfully amplified the mtDNA specific target product in all samples (Figs. 3, 4, white arrows). However, the actin and RBCL primers did not show the amplification of the cpDNA and nDNA specific target products, respectively, in most of the samples (Figs. 3, 4, red arrows). Also, in some samples (Figs. 3, 4, blue arrows), a very faint band with the actin and RBCL primers was observed, indicating the presence of some residual cpDNA and nDNA in these samples.

### Quantity and quality assessment by spectrophotometry

To determine the quantity and contamination by RNA and proteins, the extracted mtDNA was subjected to a spectrophotometric analysis. The quantity of the purified mtDNA was measured in two different ways using the PicoGreen and NanoDrop methods. These analyses confirmed large quantities of extracted mtDNA from all the assayed samples (Table 1). Similarly, RNase and proteinase K treatments of mtDNA were effective in removing RNA and proteins, respectively. The A_260_/A_280_ and A_260_/A_230_ ratios are typically used to determine the extracted DNA contamination with biological, organic and inorganic substances. The ideal A_260_/A_280_ and A_260_/A_230_ ratios for pure double-stranded DNA are generally considered to be 1.8–1.9 and 2.0–2.2, respectively [26]. Our analyses showed that A_260_/A_280_ and A_260_/A_230_ ratios for most of the samples were in the ideal range (Table 1). This result provided additional confirmation on the purity of the extracted mtDNA samples.

## Discussion

Our attempt to improve the method for isolating mitochondria from different tissues and species was fruitful, and the isolated mitochondria were successfully applied to extract mtDNA from all the assayed samples of different species. Large amount of mtDNA were obtained and the extracted mtDNA was largely free of cpDNA and nDNA contamination. The major advantage of the improved method mainly reflects its wider application with the same mitochondria grinding medium to different tissues and species, although some modification is required for specific extensions (Fig. 1). Thus, this improvement is significant with an enlarged scope of future plant mitochondria research.

One of the technical advances for the improvement is the use of a single Percoll gradient to remove chloroplasts and nuclei, instead of purifying mitochondria through continuous/discontinuous density gradients. The empirical evaluation outcomes are encouraging. For example, the testing using cpDNA and nDNA specific primers with extremely high concentration (120 ng) of the purified mtDNA as template revealed only some faint bands in some samples (Figs. 2, 3, 4). This finding helped to confirm that the extracted mtDNA had minimal contaminations from other DNAs. The faint bands were not seen when a lower amount (90 µg) of mtDNA template was used (results not shown). These results suggest that the proportion of cpDNA and nDNA contamination in mtDNA obtained from the improved method could be minimal.

We further confirmed the purity of the isolated mtDNA by spectrophotometry. Normally, a higher A_260_/A_280_ ratio indicates RNA contamination, whereas a lower value indicates protein contamination. A lower A_260_/A_230_ ratio indicates presence of phenols and carbohydrates, while a higher ratio indicates errors during blanking [27]. The ideal A_260_/A_280_ and A_260_/A_230_ ratios for pure double-stranded DNA are generally considered to be 1.8–1.9 and 2.0–2.2, respectively [26]. Our evaluations revealed an acceptable range of A_260_/A_280_ and A_260_/A_230_ ratios for most of the assayed samples (Table 1).

We were also able to isolate mitochondria and extract mtDNA from both viable and physiologically dead seeds (Fig. 3). This improvement is significant, as these isolated mitochondria and mtDNA can be used to study the differences in the mitochondria of dead and viable seeds at physiological, biochemical, and molecular levels. However, the empirical evaluation also showed that dead seeds possessed more degraded DNA when compared to normal seeds and leaf tissues (Fig. 3, green arrow). Such difference should not be surprising, as dead seeds are physiologically inactive and more degraded mtDNA may be due to DNases activity or lack of repair mechanisms. An encouraging fact is that the PCR amplifications on all the mtDNA samples were successful, suggesting that the purified mtDNA could be used for downstream PCR applications.

Given that we were able to use the improved method or its extensions for different tissues and plants, it is likely that the present method could be extended to other plants species, although some minor optimizations may be needed. It might also be useful for extracting proteins or for performing a functional analysis with the purified mitochondria, although we did not evaluate these aspects in our current assays. Little is known about the effectiveness of this method with green leaf tissues of a plant species. Mitochondria isolation from green leaves may present another technical problem due to presence of a higher amount of phenolic compounds [28]. Also, green leaf tissues are rich in chloroplasts that are difficult to remove during mitochondria preparation [29]. Future efforts are needed to optimize some of the procedures for use with green leaf tissues of an assayed species.

In spite of some limitation, the improved method should help to widen the scope of future plant mitochondria research. The isolated mitochondria from different plant samples can be used for mtDNA extraction with, minimal cpDNA and nDNA contamination. The extracted mtDNA should be suitable for high throughput sequencing and other genetic studies, including restriction, ligation, somatic transformation, and molecular marker application. Currently, we are evaluating its application to next generation sequencing for the characterization of mtDNA mutations.

## Conclusion

Here we reported an improved method for isolating plant mitochondria from dead seeds, viable seeds, and etiolated leaf tissue of several plant species. The isolated mitochondria were successfully applied to extract mtDNA from all the assayed samples. A large amount of mtDNA was obtained and the extracted mtDNA was largely free of cpDNA and nDNA contamination. The major advance in the improved method mainly reflects the wider application with the same mitochondria grinding medium to different tissues and species, although some modification is required. The improvement is significant, as it helps to widen the scope of future plant mitochondria research.

## Methods

### Methods for isolating mitochondria and extracting mtDNA

#### Improved method for isolating mitochondria from dry starchy seeds

The improved protocol was based mainly on two previously published protocols [16, 25] to isolate mitochondria from dry wheat seeds. The improved protocol uses a simple Grinding Medium (0.5 M sucrose, 1 mM EDTA, 70 mM KH_2_PO_4_, pH 7.5) with the addition of modifiers as indicated throughout the protocol. The protocol consists of the following 19 steps:

1. Select 30 healthy, mature, and dry wheat seeds.
2. Collect embryo tissue from the seeds by cutting away the endosperm with a razor blade.
3. Grind the extracted embryos with a mortar and pestle. The following steps are to be carried out at 4 °C.
4. Add 6 ml of Grinding Medium + BSA + β-ME (add 0.80 % BSA, and 0.1 % β-mercaptoethanol, just before use, to the Grinding Medium pre-chilled to 4 °C) and continue grinding until a homogeneous mixture is formed.
5. Transfer the homogenate into a 15 ml Falcon tube and centrifuge at 1800*g* for 7 min to pellet nuclei, chloroplasts, and cell debris.
6. Transfer the supernatant to a new 15 ml tube and spin at 20,000*g* for 17 min or 17,200*g* for 20 min.
7. Discard the supernatant and carefully resuspend the pellet by adding 2 ml of Grinding Medium + BSA + β-ME and slowly pipette up and down several times. Divide the supernatant evenly between to two new 1.5 ml Eppendorf tubes. (A portion of the resuspended pellet could be used to extract unpurified mtDNA as positive control).
8. Centrifuge the suspension at 1800*g* for 7 min to pellet remaining nuclei, chloroplasts, and cell debris.
9. Transfer the supernatant to a new 1.5 ml Eppendorf tube and discard the pellet. Steps S8 and S9 are repeated a total of two times.
10. Transfer the supernatant to a new 1.5 ml Eppendorf tube and discard the pellet. Centrifuge the supernatant at 4000*g* for 20 min to pellet residual chloroplasts.
11. Centrifuge the supernatant at 20,000*g* for 17 min or 17,200g for 20 min to pellet mitochondria.
12. Discard the supernatant and keep the pellet on ice during subsequent purification steps.
13. In the subsequent steps use prechilled Grinding Medium + BSA (add 0.8 % BSA to Grinding Medium just before use).
14. Carefully resuspend and recombine the two pellets by adding a total of 300 µl of Grinding Medium + BSA and slowly pipette up and down several times.
15. Centrifuge the suspension at 2000*g* for 5 min and transfer the supernatant to a new 1.5 ml tube. Discard the pellet.
16. Centrifuge the supernatant at 20,000*g* for 17 min or 17,200*g* for 20 min.
17. Discard the supernatant and resuspend the resulting pellet by adding 1 ml of Grinding Medium + BSA and slowly pipetting up and down several times.
18. Centrifuge the suspension a second time at 20,000*g* for 17 min or 17,200*g* for 20 min.
19. Discard the supernatant and keep the pellet on ice for mtDNA extraction using the extraction method below.

#### Method for extracting mtDNA from isolated mitochondria

1. For each pellet, prepare 20 µl of buffer RDD and 10 µl of DNase I stock solution from RNase-free DNase set (Qiagen, cat. no. 79254). Resuspend the pelleted mitochondria in 175 µl of Grinding Medium + BSA (S13) by slow pipetting up and down. Add 30 µl of RDD + DNase I and gentle mix by inversion. Incubate the suspension at 30 °C for 30 min.
2. Centrifuge the suspension at 17,200*g* for 20 min and discard the supernatant.
3. Extract mtDNA using the QIAamp DNA mini kit (Qiagen, cat. no. 51304) “DNA Purification from Tissues” protocol (Third Edition, June 2012) starting with the addition of 80 µl of PBS and 100 µl ATL buffers (step 2c, page 33) to the pelleted mitochondria and proceeding directly to step 3 of the mini kit protocol.
4. During mtDNA extraction, proteinase K (supplied with kit) and RNase A (Qiagen, cat. no. 19101) treatments are performed according to instructions in the supplied kit handbook.

#### Extension for dead or viable seeds

This extension was originally developed for dead (D) or viable (V) wheat seeds. Mitochondria isolation from dead seeds proceeds first with D1–D4, followed by DV1–DV7; similarly, the extension for seeds known to be viable starts first with V1–V3 and then proceeds to DV1–DV7.

1. Collect 35 dead wheat seeds (those which did not germinate) into a 50 ml Falcon tube after a germination test.
2. Add approximately 30 ml of 95 % ethanol to the seeds and gently shake for 2 min to remove contaminating fungus produced from the germination test. Carefully discard the ethanol and avoid any seed loss.
3. Repeat step D2, 1 or 2 additional times (depending upon the fungus infection) until seeds visually become clear of fungus.
4. Wash the seeds for 1 min 6–8 times with approximately 30 ml of autoclaved distilled water and dry on blotting paper or paper towel.
5. Select 25 viable wheat seeds (abnormally or normally germinated) after a germination test.
6. Prepare embryo tissue from these seeds by first removing and discarding the seedling root and shoot then gently squeeze the seed to eject the remaining embryo tissue.
7. Rinse the isolated embryo tissue with Grinding Medium + BSA + β-ME (S4) to remove any excess starch and contaminating fungus produced from the germination test.
  1. Grind the dead seeds or the washed embryo tissue from viable seeds with a mortar and pestle and follow steps S3–S14 for dry wheat seeds as described above. After completing step S14 follow the remaining procedures below.
  2. Carefully layer the 300 µl suspension from step S14 on top of 700 µl of 7 % Percoll (prepared with Grinding Medium + BSA from step S13) in a 1.5 ml Eppendorf tube and centrifuge at 2000*g* for 5 min.
  3. Without disturbing the pellet, carefully remove the supernatant to a new 1.5 ml Eppendorf tube.
  4. Add 400 µl of Grinding Medium + BSA to the supernatant and gently mix by slowly inverting the tube 5–7 times.
  5. Centrifuge the suspension at 2000*g* for 5 min and transfer the supernatant to a new 1.5 ml tube. Discard the pellet.
  6. Centrifuge the supernatant at 20,000*g* for 17 min or 17,200*g* for 20 min.
  7. After step DV6 continue with steps S17–S19 to purify the mitochondria and then apply steps M1–M4 to extract mtDNA.

#### Extension for etiolated leaf tissue

This extension was developed to isolate mitochondria from etiolated wheat leaf tissue. The protocol is described below.

1. Collect approximately 2 g of etiolated wheat leaf tissue from abnormally or normally germinating seeds by cutting away seed and other unwanted tissues with a razor blade.
2. Grind the leaf tissue with a mortar and pestle then follow steps S3–S14. Use Grinding Medium + BSA + β-ME + PVP (Grinding Medium with 0.80 % BSA, 0.1 % β-mercaptoethanol, and 0.6 % PVP) in step S4 and filter the homogenate through Miracloth (Calbiochem, cat. no. 475855, Darmstadt, Germany) before going to step S5. After completing step S14 follow the remaining procedures below.
3. Carefully layer the 300 µl suspension from step S14 on top of 700 µl of 7 % Percoll (prepare using Grinding Medium + BSA from step S13) in a 1.5 ml Eppendorf tube and centrifuge at 2000*g* for 5 min.
4. Without disturbing the pellet, carefully remove the supernatant to a new 1.5 ml Eppendorf tube.
5. Centrifuge the supernatant at 20,000*g* for 17 min or 17,200*g* for 20 min and discard the resulting supernatant.
6. Carefully resuspend the pellet by adding 300 µl of Grinding Medium + BSA (S13) and slowly pipette up and down several times. Repeat steps L3–L4 an additional time.
7. Add 400 µl of Grinding Medium + BSA (S13) to the supernatant and gently mix by slowly inverting the tube 5–7 times.
8. Centrifuge the suspension at 2000*g* for 5 min and transfer the supernatant to a new 1.5 ml tube. Discard the pellet.
9. Centrifuge the supernatant at 20,000*g* for 17 min or 17,200*g* for 20 min.
10. After step L9, continue with steps S17–S19 to purify the mitochondria and then apply steps M1–M4 to extract mtDNA.

#### Extension for oily seeds

This extension was originally developed to isolate mitochondria from oily seeds of three other plant species (Arabidopsis, cultivated flax, and yellow mustard).

1. Select whole, mature, and dry Arabidopsis (500 mg), flax (20), or mustard (50) seeds.
2. Grind the seeds with a mortar and pestle then follow steps S3–S14. Use Grinding Medium + BSA + β-ME + Sorbitol (Grinding Medium with 0.80 % BSA, 0.1 % β-mercaptoethanol, and 0.7 M sorbitol). After completing step S14 follow the remaining procedures below.
3. Carefully layer the 300 µl suspension from step S14 on top of 700 µl of 7 % Percoll (prepared using Grinding Medium + BSA from step S13) in a 1.5 ml Eppendorf tube and centrifuge at 2000*g* for 5 min.
4. Without disturbing the pellet, carefully remove the supernatant to a new 1.5 ml Eppendorf tube. Add 400 µl of Grinding Medium + BSA (S13) to the supernatant and gently mix by slowly inverting the tube 5–7 times.
5. Centrifuge the suspension at 2000*g* for 5 min and transfer the supernatant to a new 1.5 ml tube. Discard the pellet.
6. Centrifuge the supernatant at 20,000*g* for 17 min or 17,200*g* for 20 min.
7. After step O7, continue with steps S17–S19 to purify the mitochondria and apply steps M1–M4 to extract mtDNA.

### Empirical evaluation of the improved method

#### Tissue preparation and selection

To validate the improved method for the isolation of mitochondria from dry seeds, wheat var. AC Barrie and Superb and cultivated oat var. CDC Dancer were selected and their mature, healthy, and dry starchy seeds were used for mitochondria isolation and then mtDNA extraction.

**Fig. 5:**
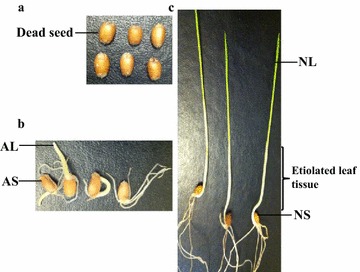
Presentation of five wheat seed and leaf tissues used for the extraction of mtDNA from two wheat genotypes. Healthy seeds of AC Barrie and Superb were exposed to an accelerated ageing test (AAT) for 72 h at 43 °C. Following AAT, a germination test was performed and five seed and leaf tissues were collected for mtDNA extraction: dead seeds in the panel **a**; abnormally germinated seeds tissue (AS) and abnormally germinated leaf tissue (AL) in the panel **b**; normally germinated seeds tissue (NS) and normally germinated leaf tissue (NL) in the panel **c**

To assess the extensions to dead seeds, viable seeds and etiolated leaf tissue, AAT and germination tests were performed with the two wheat varieties. The AAT was performed using the inner chamber ‘tray method’ following the set-up as described [30]. Approximately 100 healthy and mature seeds of two wheat varieties were evenly placed on screened frame of AAT boxes in three replicates. In the AAT box, 40 ml of ddH_2_O were added and screened frame was placed on top of it. Controlled environment incubator (Hoffman Manufacturing Inc., Jefferson, OR, USA) was pre-warmed to 43 °C for 24 h. AAT boxes were placed in the chamber for 72 h according to the suggested AAT guidelines [30]. Following AAT, germination test was performed following the genebank operational guidelines [31]. For each replication, three germination papers were soaked in 47 ml distilled water. The acrylic seed spacer (Hoffman Manufacturing Inc., Jefferson, OR, USA) was used to spread 100 seeds on the paper (two stacked together). The third paper was placed on top of the stacked papers with seeds. The three papers were folded from the bottom and rolled. The rolls were placed in plastic bag. The plastic bag was placed in incubator at 20 °C with 8 h light and 16 h dark. Germination rates were observed on the 7th day. Following the germination tests, three types of seeds were identified from both wheat varieties (Fig. 5). They were (1) dead seeds for which no sign of germination was observed; (2) abnormally germinated seeds for which either root or shoot grew a few millimeters or less; and (3) normally germinated seeds for which normal root and shoot growth was observed. Normally and abnormally germinated seeds were considered as viable seeds for this evaluation. In total, five seed and leaf tissues were collected for mitochondria and mtDNA extractions: dead seeds; abnormally germinated seeds tissue (AS); abnormally germinated leaf tissue (AL); normally germinated seeds tissue (NS); and normally germinated leaf tissue (NL). The collected dead seeds, viable seeds (AS, NS) and leaf tissues (AL, NL) were used to assess the extensions to dead seeds, viable seeds and leaf tissues, respectively.

To evaluate the extension to oily seeds, other three plant species (Arabidopsis var. Columbia; flax var. CDC Neela; and yellow mustard var. AC Pennant) were also arbitrarily selected and their mature, healthy, and dry oily seeds were used for empirical evaluation. These assayed seeds and those wheat and oat seeds (Table 1) were obtained from the related germplasm collections maintained at the Plant Gene Resources of Canada.

#### Mitochondria isolation and mtDNA extraction

The selected seed and/or leaf tissues were used to isolate mitochondria and extract mtDNA according to the improved method or it specific extensions as described above. Each isolation and extraction was performed at least twice to assess the consistency in each application.

#### Quantity and quality assessments

Quantity and quality of the extracted mtDNA were assessed using NanoDrop 8000 (Thermo Scientific). DNA purity was determined by calculating the absorbance ratios A_260_/A_280_ and A_260_/A_230_. mtDNA concentration was also measured by Quant-iT™ PicoGreen^®^ dsDNA assay kit (Life Technologies, cat. no. P7589, Eugene, OR, USA). Samples measured by NanoDrop were diluted to 40 ng/µl and used for quantity assessment by PicoGreen according to manufacturer’s instructions.

#### PCR assessment of mtDNA purity

The purity of the extracted mtDNA was analyzed by PCR amplification using mitochondrial, COX1; nuclear, actin; chloroplast, RBCL genes specific primers (see Additional file 1). PCRs were performed on C1000 Thermal Cycler (Bio-Rad) in 20 µl volumes containing 120 ng mtDNA, 2 µl of 10X standard *Taq* reaction buffer (New England Bio Labs, cat. no. B9014S, Whitby, ON, Canada), 0.1 mM dNTPs, 0.25 pM primers (forward and reverse each), 0.3 µl of *Taq* polymerase (New England Bio Labs, cat. no. M0273X, Whitby, ON, Canada) and ddH_2_O to make volume to 20 µl. Reaction conditions were as follow: 95 °C for 5 min followed by 30 cycles at 95 °C for 30 s, 55 °C (actin) or 60 °C (COX1, RBCL) for 30 s and 72 °C for 1 min and a final extension at 72 °C for 7 min.

To visualize PCR products for purity and quality, agarose gel electrophoresis was applied. Approximately 28 µg of purified mtDNA or PCR reaction from each sample were loaded onto 1.5 % agarose gel. The electrophoresis was carried out in 1x TAE buffer (40 mM Tris-Acetate, 1 mM EDTA) at 100 V and 3 mAh for 3 h. The gels were visualized by staining with 0.5 mg/mL ethidium bromide.

## Additional file

10.1186/s13007-015-0099-x List of primers used in this study, along with target genes, symbols and primer sequences.

## Abbreviations

AAT: accelerated ageing test
AL: abnormally germinated leaf tissue
AS: abnormally germinated seed tissue
COX1: cytochrome C oxidase I
cpDNA: chloroplast DNA
mtDNA: mitochondrial DNA
nDNA: nuclear DNA
NL: normally germinated leaf tissue
NS: normally germinated seed tissue
PVP: polyvinylpyrrolidone
RBCL: rubisco-large subunit
