# Supplementary material for: An improved method with a wider applicability to isolate plant mitochondria for mtDNA extraction
Source: Plant Methods. 2015 Dec 21;11:56. doi: 10.1186/s13007-015-0099-x (PMC4687074; doi:10.1186/s13007-015-0099-x)
Supplement: Supplementary file 1 — 10.1186/s13007-015-0099-x List of primers used in this study, along with target genes, symbols and primer sequences. [file 13007_2015_99_MOESM1_ESM.docx]

**Additional files**

Zaheer Ahmed and Yong-Bi Fu (2015) An improved method with a wider applicability to isolate plant mitochondria for mtDNA extraction. Plant Methods

**Additional file 1** List of primers used in this study, along with target genes, symbols and primer sequences.

| Organelle | Gene | Symbol | Forward (5' to 3') / Reverse (5' to 3') |
| --- | --- | --- | --- |
| Mitochondrion | Cytochrome C oxidase I | COX1 | TTTCATCTTCGGTGCCATTGCAGG |
|  |  |  | /ACCTATCATCGCCGGCATAACCAT |
| Chloroplast | Rubisco- large subunits | RBCL | ACGATGCTATCACATCGAGCCTGT |
|  |  |  | /AACGTAGAGCACGTAGGGCTTTGA |
| Nucleus | Actin | Actin | GAATCCATGAGACCACCTAC |
|  |  |  | /AATCCAGACACTGTACTTCC |

Primer source: COX1 = 178 bp (designed by Mr. Gregory Peterson, Plant Gene Resources of Canada), RBCL = 158 bp (designed by Mr. Gregory Peterson), and actin = 206 bp (Paolacci AR, Oronzo AT, Enrico P, Mario C. Identification and validation of reference genes for quantitative RT-PCR normalization in wheat. BMC Mol Biol. 2009; 10:11).
